# Skin Mycobiota of the Captive Giant Panda (*Ailuropoda melanoleuca*) and the Distribution of Opportunistic Dermatomycosis-Associated Fungi in Different Seasons

**DOI:** 10.3389/fvets.2021.708077

**Published:** 2021-11-04

**Authors:** Xiaoping Ma, Gen Li, Yaozhang Jiang, Ming He, Chengdong Wang, Yu Gu, Shanshan Ling, Sanjie Cao, Yiping Wen, Qin Zhao, Rui Wu, Zhicai Zuo, Zhijun Zhong, Guangneng Peng

**Affiliations:** ^1^Key Laboratory of Animal Disease and Human Health of Sichuan Province, College of Veterinary Medicine, Sichuan Agricultural University, Chengdu, China; ^2^Bioengineering Department, Sichuan Water Conservancy Vocational College, Chengdu, China; ^3^China Conservation and Research Center for the Giant Panda, Chengdu, China; ^4^College of Life Sciences, Sichuan Agricultural University, Chengdu, China

**Keywords:** giant panda (*Ailuropoda melanoleuca*), dermatomycosis, fungi, skin mycobiota, seasonality

## Abstract

Dermatomycosis is the second major cause of morbidity in giant pandas (*Ailuropoda melanoleuca*), and seriously endangers its health. Previous observations indicated that the occurrence of dermatomycosis in the giant panda varies in different seasons. The skin microbiota is a complex ecosystem, but knowledge on the community structure and the pathogenic potentials of fungi on the skin of the giant panda remains limited. In this study, samples from the giant panda skin in different seasons were collected, and the mycobiota were profiled by 18S rRNA gene sequencing. In total, 375 genera in 38 phyla were detected, with Ascomycota, Basidiomycota, Streptophyta, and Chlorophyta as the predominant phyla and *Trichosporon, Guehomyces, Davidiella, Chlorella, Asterotremella*, and *Klebsormidium* as the predominant genera. The skin mycobiota of the giant panda changed in the seasons, and the diversity and abundance of the skin fungi were significantly higher in spring, autumn, and summer than in the winter. Several dermatomycosis-associated fungi were detected as opportunists in the skin mycobiota of healthy giant pandas. Clinical dermatomycosis in the giant panda is observed more in summer and autumn. In this study, the results indicated that the high diversity and abundance of the skin fungi may have enhanced the occurrence of dermatomycosis in autumn and summer, and that dermatomycosis-associated fungi are the normal components of the skin mycobiota.

## Introduction

Dermatomycosis is the second cause of morbidity in giant pandas (*Ailuropoda melanoleuca*), after gastroenteritis, and seriously endangers their health (1, 2). Animal and human skin is a complex ecosystem, colonized by numerous microbes of various beneficial or pathogenic potentials (3). It is a critical interface between the body and its external environment, which prevents the loss of moisture and blocks the entry of pathogenic organisms (4). Dermatomycosis is challenging to treat due to the extended duration of treatment required, with short-term treatment unlikely to achieve resolution. It damages the skin and hair of giant pandas and, therefore, affects the growth and appearance of the hair coat (5). Dermatomycosis can even compromise the immunity of giant pandas and subsequently increase the morbidity and mortality from other diseases (6). Several fungi have been found to be conditionally associated with dermatomycosis of giant pandas, including *Candida* spp., *Cladosporium cladosporioides, Microsporum gypseum, Mucor* spp., *Trichophyton mentagrophytes*, and *Trichosporon* spp. (5, 7–11). However, sufficient knowledge on the community structure and the pathogenic potentials of fungi on the skin of the giant panda remains lacking.

Captive breeding is an effective approach to protecting giant panda populations but requires close housing of these individuals. At the China Conservation and Research Center for Giant Pandas (Ya'an, China), dermatomycosis was found more likely for captive giant panda to develop in spring, summer, and autumn than in winter. As fungal pathogens are mostly opportunistic (12), it is important to determine the skin mycobiota of the giant panda to guide the efficient control of dermatomycosis. In addition, it is unknown how the skin mycobiota of the captive giant panda changes, and if an association exists between the skin mycobiota and the dermatomycosis in different seasons. Culture-dependent methods have been applied to characterize the fungal community of the giant panda skin. However, only several fungal species were cultured successfully (5, 7–11). With the advancements in next-generation sequencing and bioinformatics, culture-independent method (ribosomal DNA sequencing) has been widely used to characterize the mycobiota of both humans and animals (13–15). In this study, to determine the skin mycobiota of the captive giant panda in different seasons, samples from the skin of the giant panda in four seasons in 1 year were collected, and the mycobiota were profiled by 18S rRNA gene sequencing.

## Materials and Methods

### Sample Collection

Samples were collected from clinically healthy giant pandas (five females and four males) at the China Conservation and Research Center for Giant Pandas (Ya'an, China). The pandas were housed in dozens of independent enclosures on a mountain where a similar environment to wild pandas was preserved with heavy broad-leaved forests, green bamboos, and thorns. Each enclosure included an open outdoor area and a closed indoor area, and housed one or two giant pandas. They were free to move around indoors and outdoors in their own enclosure but barely met pandas from other enclosures. They were fed with a diet of about 10% steamed cornbread and fruits and 90% bamboo shoots, and were allowed to drink water *ad libitum* (16). The sampled pandas were conditioned to human presence and did not require restraint or anesthesia for sample collection but cooperated with the procedure during feeding. Samples were collected from the skin of the proximal dorsal thoracic limb, head, or dorsum in an area of approximately 5.0 × 5.0 cm. The hair and dander on the surface were removed first by hand shears to trim most of the distal part of the hair, and then the residual hair from the prepared area was collected by sterile scalpel blade scraping and put into a 20-ml sterile tube. The sampling spots were located on the front part of the panda body and avoided the very surface layer of the skin, which minimized the artificial impacts of the environment on the skin microbiota. The personnel for sampling wore sterile protective clothing, hats, masks, and latex gloves. Samples were then transferred within 2 min of collection into a sterile plastic sample bag, shipped to the laboratory on ice within 2 h, and stored there in a −80°C freezer (16). Sampling was performed in March, June, September, and December to represent the four seasons (spring, summer, autumn, and winter, respectively). A total of 36 samples were collected (Supplementary Table 1).

### DNA Extraction, PCR, and NGS Sequencing

Total genomic DNA from the samples was extracted using the CTAB/SDS method (17). The V4 regions of 18S rRNA genes for all the 36 samples were amplified with the specific primers (18S V4: 528F:5′-GCGGTAATTCCAGCTCCAA-3′, 706R:5′-AATCCRAGAATTTCACCTCT-3′), using Phusion® High-Fidelity PCR Master Mix kit (New England Biolabs, Ipswich, MA, USA). Sequencing libraries were generated and barcoded using TruSeq® DNA PCR-Free Sample Preparation kit (Illumina, San Diego, CA, USA) following the instructions of the manufacturer. The library quality was assessed using the Qubit@ 2.0 Fluorometer (Thermo Scientific, Waltham, MA, USA) and Agilent Bioanalyzer 2100 system (Agilent Technologies, Santa Clara, CA, USA). The amplicons were sequenced on an IlluminaHiSeq2500 platform with 250-bp paired-end reads.

### Data Analysis

Raw reads were preprocessed to remove the adapters and low-quality reads by the following procedures according to the QIIME tag quality control process (18). Filtered reads pairs were merged using FLASH (V1.2.7, http://ccb.jhu.edu/software/FLASH/) (19). OTUs were assigned at 97% sequence similarity by Uparse software (Uparse v7.0.1001, http://drive5.com/uparse/) (20) and annotated against the Silva Database (http://www.arb-silva.de/) (21) using RDP classifier algorithm (version 2.2, http://sourceforge.net/projects/rdp-classifier/) (22). Subsequent analysis of alpha diversity and beta diversity were all performed using QIIME2 and displayed with R software (Version 3.6.3) and GraphPad Prism7. Linear discriminant analysis coupled with effect size (LEfSe) was performed to identify the fungal taxa differentially represented between seasons at genus (23).

## Results

### Overview of the Sequencing Data

The amplicons of 18S rDNA V4 region of 36 samples were sequenced by the Illumina platform. After quality filtering, a total 1,992,224 reads of 18S rDNA gene were obtained, and the reads for each sample were between 46,691 and 65,374. The filtered reads were then subjected to classification of the fungal taxa. After singleton OTUs were removed, a total of 1,195 OTUs were obtained in the samples, and 490–842 OTUs were identified for each sample. The samples were then rarefied to 46,691 reads for subsequent analysis of alpha diversity and beta diversity. The OTU numbers were calculated along with rarefaction curves (Supplementary Figure 1). The rarefaction curves started to be flat at 7,677 reads sampling. The results indicated that sequencing depth was sufficient to represent the diversity in each sample.

### Skin Mycobiota of the Giant Panda Changed in Different Seasons

**Figure 1:**
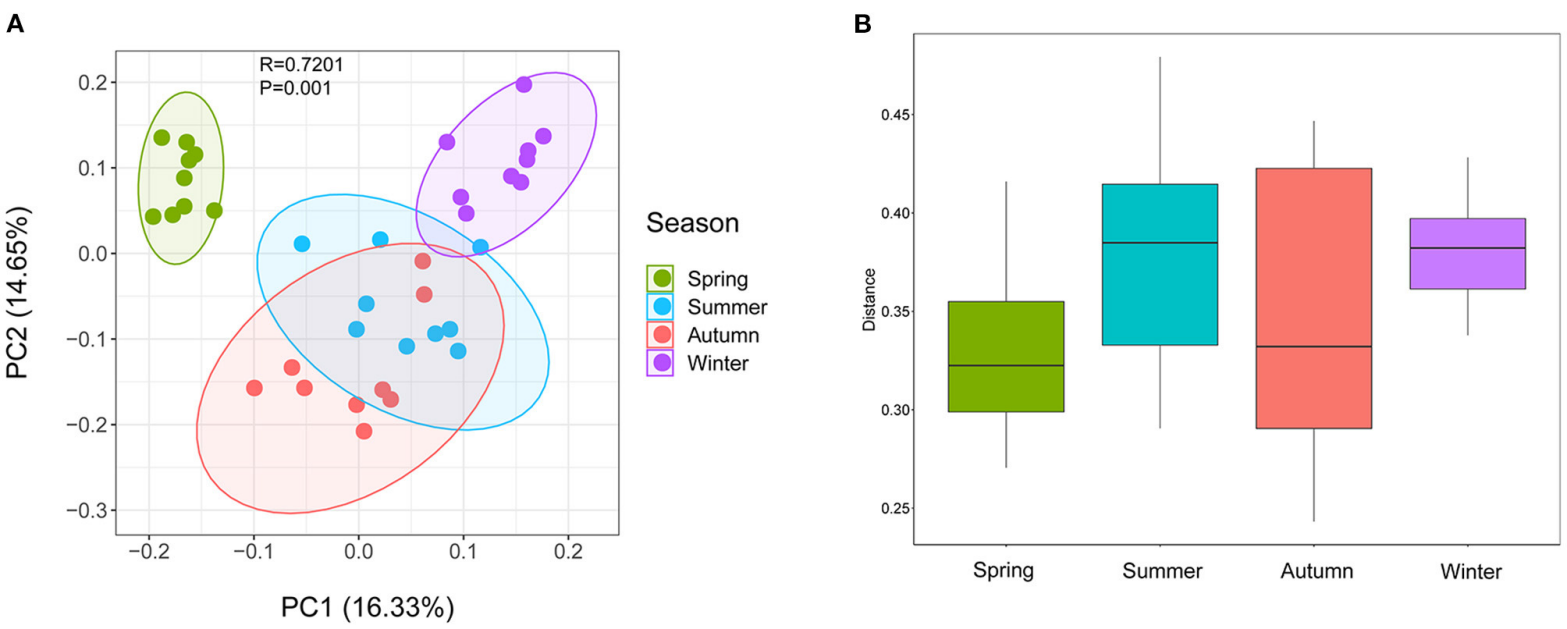
Beta diversity of dermatophyte microbiota of the giant panda (*Ailuropoda melanoleuca*) from different seasons. **(A)** Principal coordinate analysis based on unweighted UniFrac metrics indicates that dermatophyte microbiota of the giant panda (*A. melanoleuca*) are associated with the seasons (ANOSIM: R = 0.7201, *p* = 0.001). The close clustering of the samples from each season demonstrates the high phylogenetic similarities of their microbiota. **(B)** PERMANOVA analysis of the UniFrac distances for the samples between each season shows that the season is significantly different from each other (*p* < 0.01).

**Figure 2:**
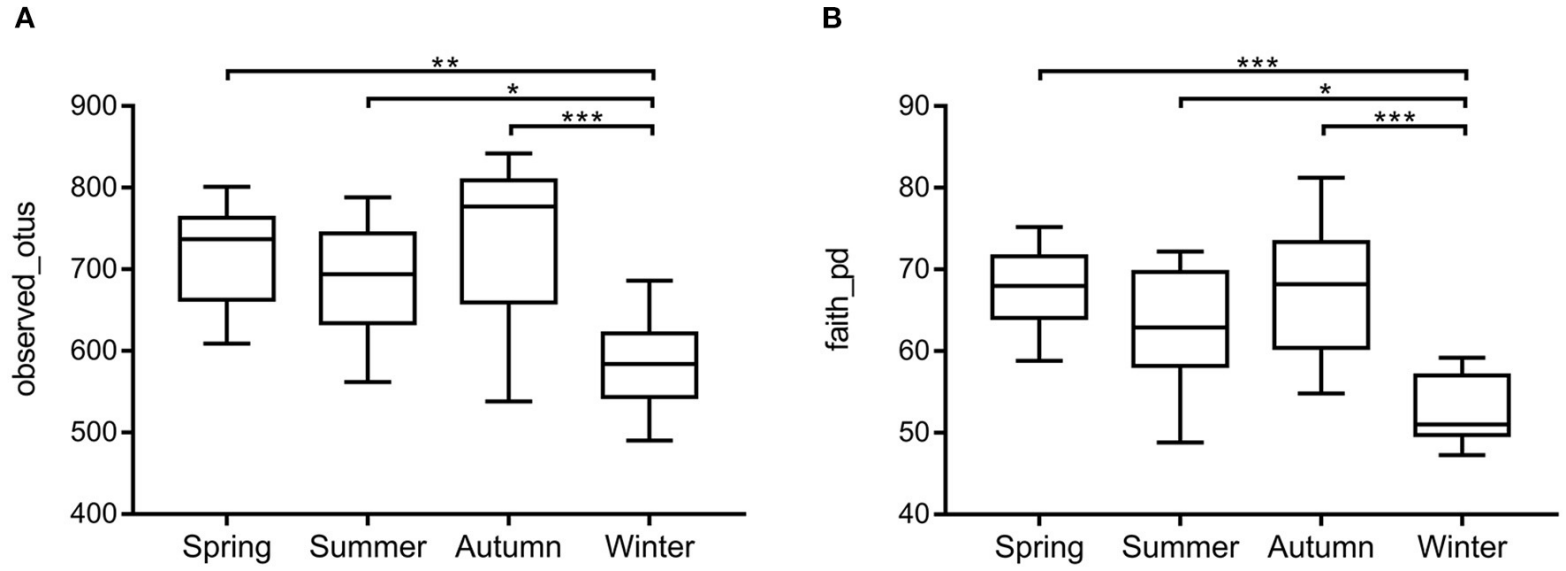
Species richness and phylogenetic diversity of dermatophyte microbiota of the giant panda (*A. melanoleuca*) measured by 18S rDNA sequencing. Comparison of alpha diversity between seasons are shown in **(A)** (observed_otus) and **(B)** (faith_pd), respectively. The differences are not significant between the samples from autumn, spring, and summer, but the samples from those three seasons are all significantly different from the samples from winter (*p < 0.05, **p < 0.01, ***p < 0.001).

Samples were grouped by beta diversity matrix. Based on the unweighted UniFrac distance metrics of beta diversity, PCoA analysis revealed that the samples clustered together according to seasons (Figure 1A). PERMANOVA analysis of the sample distances between different seasons showed that the skin mycobiota of each season was significantly different from each other (*p* < 0.01) (Figure 1B), indicating that the skin mycobiota of the giant panda are different in the seasons. The species diversity of the samples from each season was compared according to the alpha diversity index (observed_otus, faith_pd) (Figures 2A,B). The observed OTUs were highest in samples from autumn, followed by spring, summer, and winter (Figures 2A,B). However, the differences were not significant between the samples from spring, summer, and autumn, but the samples from those three seasons were all significantly different from the samples from winter (Figures 2A,B). The results indicated that species diversity in winter was significantly lower than that from the other seasons.

### The Overall Fungal Community Structure

**Figure 3:**
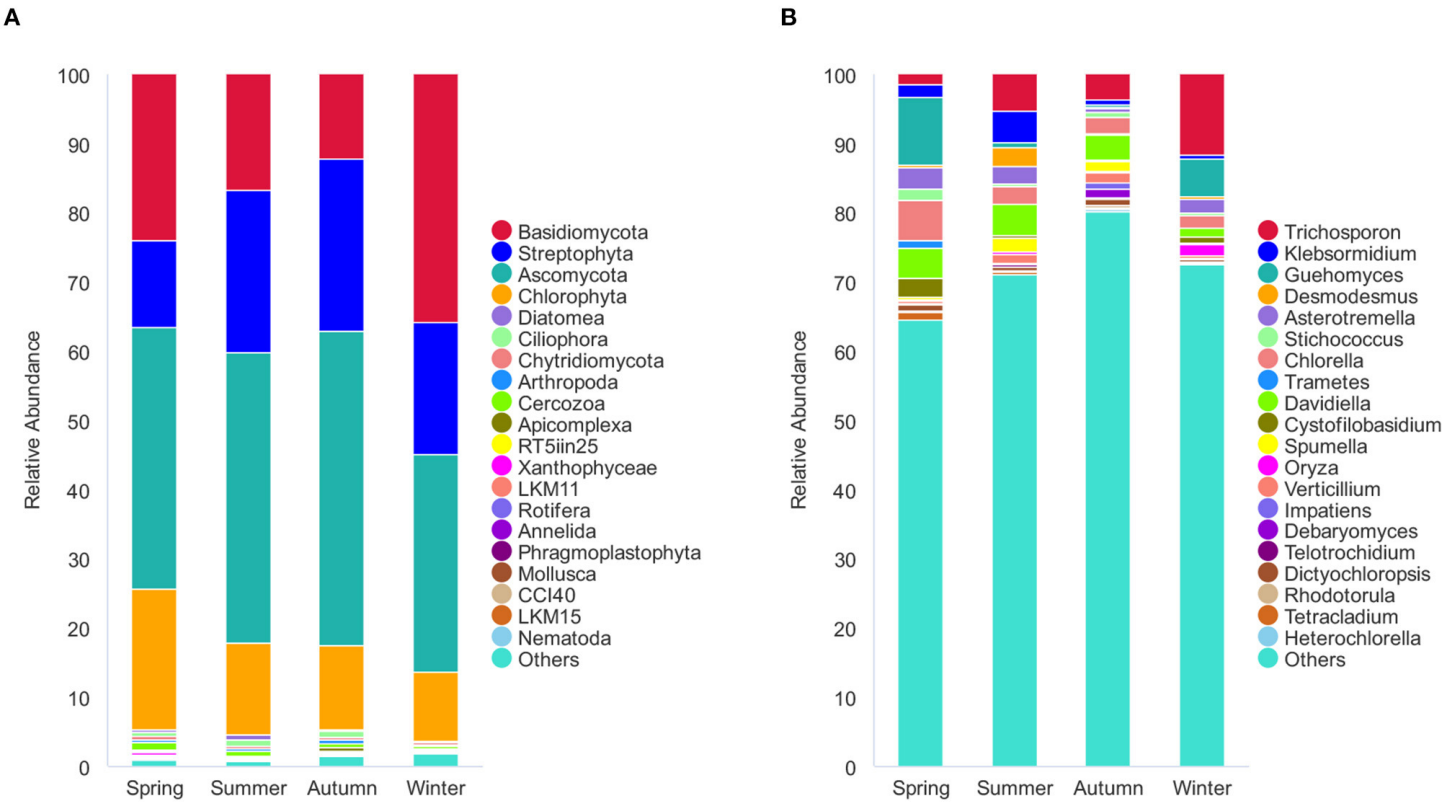
Stacked bar plots showing average percentage of dermatophyte populations of the giant panda (*A. melanoleuca*) from different seasons. **(A)** Dermatophyte composition at the phylum levels (38 taxa), but only the names of the top 20 abundant taxa are listed along with the plot. **(B)** Dermatophyte composition at the genus levels (375 taxa), but only the names of the top 20 abundant taxa are listed along with the plot.

The 1,195 OTUs from all the 36 samples were classified into 38 phyla, 85 classes, 178 orders, 233 families, and 375 genera (Supplementary Table 2). At the phylum level, 95.9% OTUs were classified with definitive phylum taxa (38 phyla). Among them, Ascomycota (37.3%), Basidiomycota (21.1%*)*, Streptophyta (19%), and Chlorophyta (13.2%) were the predominant taxa (>1%) on the average of all samples (Figure 3A). At the genus level, however, only 40.5% OTUs could be classified with definitive genus (375 genera) (Figure 3B). Among them, *Trichosporon* (4.7%), *Guehomyces* (3.5%), *Davidiella* (2.9%), *Chlorella* (2.7%), *Asterotremella* (1.7%), and *Klebsormidium* (1.6%) were the predominant genera (>1%) on the average of all samples. The relative abundance of the taxa varied in different seasons. The dominant populations at the phylum and genus level are shown in Tables 1, 2. Interestingly, besides the dominant fungal taxa, some non-fungal taxa were detected in the skin microbiota although with low abundance (<1%), such as the phylum Arthropoda, Nematoda, Rotifera, Annelida, Ciliophora, Mollusca, and Apicomplexa (Figure 3A).

**Table 1:** The relative abundance of dominant fungi at the phylum level in different seasons that are more than 1%.

**Spring**	**Summer**	**Autumn**	**Winter**
Ascomycota (35.3%)	Ascomycota (39.2%)	Ascomycota (44.1%)	Ascomycota (30.4%)
Basidiomycota (22.4%)	Basidiomycota (15.7%)	Basidiomycota (11.8%)	Basidiomycota (34.6%)
Chlorophyta (19.0%)	Chlorophyta (12.5%)	Chlorophyta (11.7%)	Chlorophyta (9.6%)
Streptophyta (11.9%)	Streptophyta (21.8%)	Streptophyta (24.2%)	Streptophyta (18.3%)

**Table 2:** The relative abundance of dominant fungi at the genera level in different seasons that are more than 1%.

**Spring**	**Summer**	**Autumn**	**Winter**
*Guehomyces* (8.0%)	*Trichosporon* (4.5%)	*Davidiella* (3.3%)	*Trichosporon* (9.9%)
*Chlorella* (4.7%)	*Davidiella* (3.8%)	*Trichosporon* (3.2%)	*Guehomyces* (4.7%)
*Davidiella* (3.5%)	*Klebsormidium* (3.7%)	*Chlorella* (2.2%)	*Chlorella* (1.6%)
*Asterotremella* (2.5%)	*Asterotremella* (2.2%)	*Verticillium* (1.3%)	*Asterotremella* (1.6%)
*Cystofilobasidium* (2.2%)	*Desmodesmus* (2.2%)	*Spumella* (1.3%)	*Oryza* (1.5%)
*Klebsormidium* (1.6%)	*Chlorella* (2.1%)	*Debaryomyces* (1.2%)	
*Stichococcus* (1.4%)	*Spumella* (1.7%)		
*Leucosporidium* (1.3%)	*Verticillium* (1.1%)		
*Trichosporon* (1.1%)			

### Season-Related Genera and Abundance Variations of the Dermatomycosis-Associated Fungi

**Figure 4:**
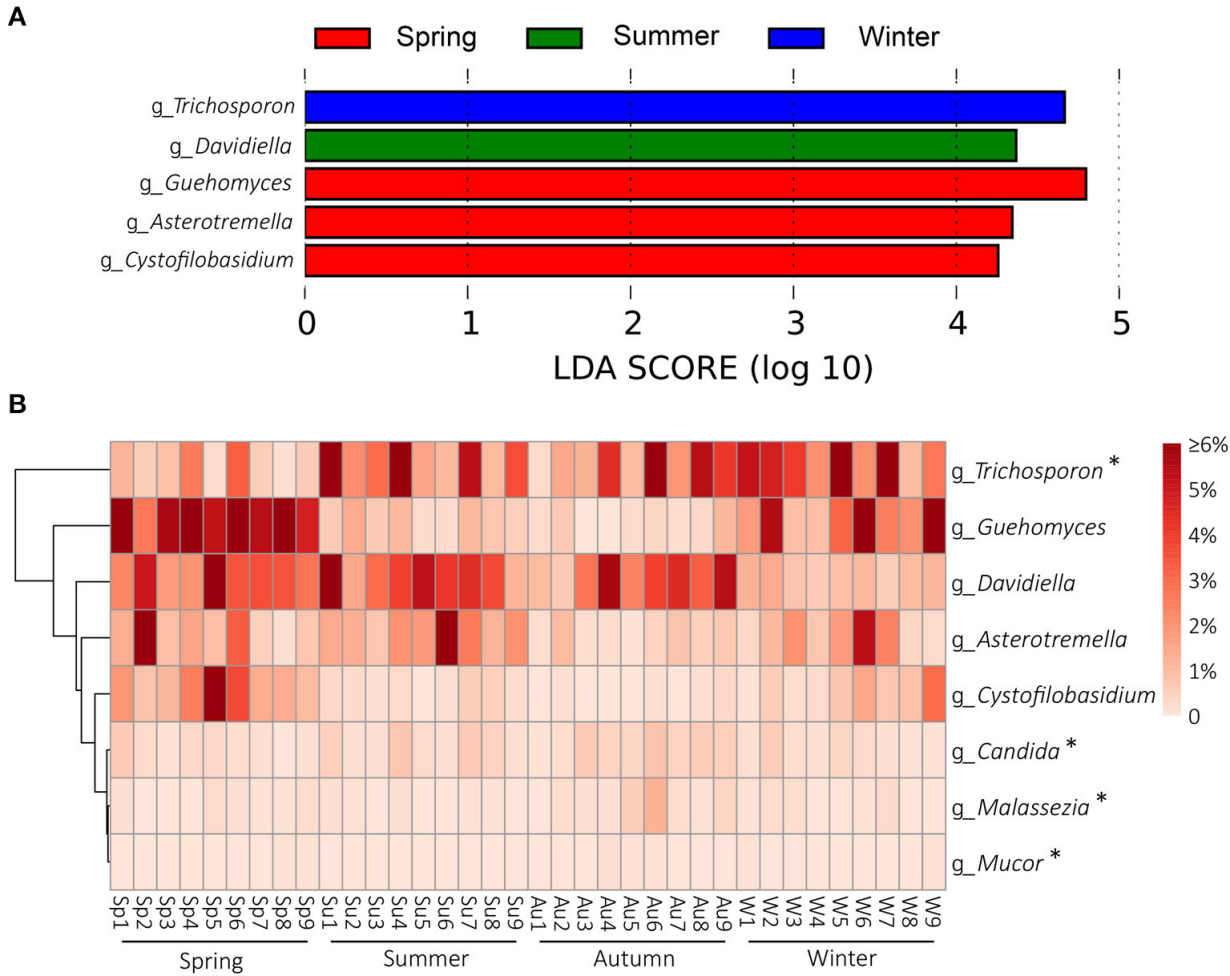
Season-related genera and abundance variation of dermatomycosis-associated fungi. **(A)** Dermatophyte genera associated with seasons identified by linear discriminant analysis coupled with effect size (LEfSe) using the default parameters. **(B)** Heatmap shows the relative abundance of season-related genera and dermatomycosis-associated fungi (only taxa with defined genus are shown; genera indicated by “*” mean the taxa previously reported to be associated with dermatomycosis of the giant panda (*A. melanoleuca*).

Season-related genera were identified by LEfSe [linear discriminant analysis (LDA) effect size] analysis. Five defined genera were associated with spring (*n* = 3), summer (*n* = 1), autumn (*n* = 0), and winter (*n* = 1), respectively (Figure 4A). Among the defined taxa, *Trichosporon* was associated with winter; *Davidiella* was associated with summer; and *Guehomyces, Asterotremella*, and *Cystofilobasidium* were associated with spring (Figures 4A,B). Several fungi have been found to be opportunistic but associated with dermatomycosis of the giant panda, including *Candida* spp., *Cladosporium* spp., *Cladosporioide* spp., *Malassezia* spp., *Microsporum gypseum, Mucor* spp., *Trichophyton mentagrophytes*, and *Trichosporon* spp. (5, 7–11). In this study, *Candida, Malassezia, Mucor*, and *Trichosporon* in the skin mycobiota from healthy giant pandas were detected (Figure 4B). *Trichosporon* was more abundant than *Candida, Malassezia*, and *Mucor*. The average relative abundance of *Trichosporon* was 4.7%, and those of *Candida, Malassezia*, and *Mucor* were 0.3, 0.2, and 0.03%, respectively. Interestingly, *Trichosporon* was season related, and it was more abundant in winter. No significant variations for *Candida, Malassezia*, and *Mucor* in different seasons were identified (Figure 4B).

## Discussion

The skin is a complex and dynamic ecosystem that is inhabited by bacteria, archaea, fungi, and viruses. The fungal inhabitants may infect the skin of the host and cause dermatomycosis (24). In this study, the skin mycobiota of the captive giant panda was profiled, and 375 genera in 38 phyla were detected. Among them, Ascomycota, Basidiomycota, Streptophyta, and Chlorophyta were the predominant phyla; and *Trichosporon, Guehomyces, Davidiella, Chlorella, Asterotremella*, and *Klebsormidium* were the predominant genera, which revealed the unique skin mycobiota of the giant panda, compared with those of other animals and humans. Previous studies, for example, showed that the canine skin was dominated by genera *Alternaria* and *Cladosporium* (25); the feline skin was dominated by genera *Cladosporium* and *Alternaria* (15); and the human skin was dominated by *Malassezia* (26). The skin mycobiota in different hosts may have been affected by genetic differences, pelage characteristics, or different hygiene practices and environmental exposures between host species (15). By beta diversity analysis, the skin mycobiota of the captive giant panda fluctuated in different seasons (Figure 1). Alpha diversity analysis showed that fungal diversity and abundance in the skin were significantly higher in spring, summer, and autumn than in winter (Figure 2). As the pandas were housed in the semi-closed enclosures, they were constantly exposed to the soil, water, and plants, in the natural outdoor environment. The development of their skin mycobiota may have been associated with the seasonal environment. Winter is cold and dry in Sichuan, China. The low temperature and dry environment in winter are not optimal for the growth and reproduction of fungi in both the environment and the skin of pandas, which may have contributed to the difference in the diversity and abundance of the skin mycobiota of the giant panda.

At the genus level, five defined taxa were significantly more abundant in different seasons. *Guehomyces, Asterotremella*, and *Cystofilobasidium* were associated with spring, *Davidiella* was associated with summer, and *Trichosporon* was associated with winter (Figures 4A,B). Isolation and physiological characterization of those fungal taxa from the environment and animals have been reported (27–31), but no insight is available on whether they are related to the skin health of animals except *Trichosporon*. *Trichosporon* is the very common fungus on the body surface of healthy giant pandas in culture-dependent isolation, and it is also an opportunistic pathogen (32). *Trichosporon* can cause superficial fungal infections such as tinea pedis, onychomycosis, and dermoid infections in humans and animals (27, 32). In a previous study, *Trichosporon* was associated with the dermatomycosis of giant panda (32). In a mouse model, it also was demonstrated that *Trichosporon* could cause dermatomycosis and develop systemic infections (32).

In addition to *Trichosporon*, several other taxa in the mycobiota of the giant panda were detected to have been opportunistically involved in dermatomycosis of humans or animals, including *Malassezia, Candida, and Mucor*, but their abundance did not change significantly in different seasons (Figure 4B). *Malassezia* is a lipophilic yeast (33), and the resident skin fungi of humans and warm-blooded animals (34). *Malassezia* often invades the cuticle of the skin and causes superficial fungal infections, such as tinea versicolor, *Malassezia* folliculitis, seborrheic dermatitis, and atopic dermatitis under suitable conditions. No cases of giant pandas infected by *Malassezia* yet have been detected. *C*. *albicans* is a very common opportunistic fungal pathogen. It exists widely in the skin, mouth, upper respiratory tract, intestinal tract, and vagina of humans and animals (35). It has been reported in an infection case of a panda cub, causing red rash on the body surface (11). *Mucor* is an important genus associated with dermatomycosis in humans and animals. It causes acute inflammation of the skin and swelling of the tissues, manifested as scleroma or plaque, purulent, necrosis, often forming eschar. Necrotic tissue may slough to form large ulcers. Mucormycosis is the third most invasive fungal infection in humans after aspergillosis and candidiasis (36). In giant pandas, *Mucor* infection could cause skin hair loss (9).

Clinically, dermatomycosis in giant panda is more common in summer and autumn. However, the results of this study did not indicate that the occurrence of dermatomycosis correlated with the abundance of dermatomycosis-associated fungi, given that many of the known associated taxa did not change their abundance with the seasons and that *Trichosporon* was even more abundant in winter (Figure 4B). It appeared that dermatomycosis-associated fungi are the normal components of the skin mycobiota in giant panda and may cause dermatomycosis conditionally. When the skin is breached by physical damage, or the host is under weak immune condition, the fungi will invade the skin and develop dermatomycosis. At the study location, it has been noticed that dermatomycosis occurs more in panda cubs and elder pandas, which may be due to their immature or compromised immune systems. The diversity and abundance of the skin mycobiota were significantly higher in spring, summer, and autumn than in winter (Figure 2). The warm and humid environment in spring summer, and autumn in Sichuan, China, may be more suitable for fungal growth and have accounted for the difference. The development of dermatomycosis is multifactorial, which may not be only associated with the fungal community structure, and also the host skin integrity, body immunity, the bacteria, virus, and ectoparasites community of the skin surfaces (37, 38). However, the high diversity and abundance of the skin fungi in autumn and summer observed in this study may have enhanced the opportunity to infect the host. In this study, besides the fungal taxa, we also detected some non-fungal taxa in the skin microbiota although with low abundance (<1%). Most of them are animal-related parasites. It remains unknown how they may be related to the skin health of the giant pandas; the “Arthropoda” found on the skin of pandas may represent samples of free-living (i.e., non-parasitic) arthropods, such as spiders or mosquitoes (39, 40). However, *Demodex* mite and Sarcoptic mite in Arthropoda may cause intensive itching and pruritus, which elicits scratch of the panda resulting in the skin damage and indirectly increase the risk of fungal infections (41, 42). Interestingly, we also found Apicomplexa on the skin. Apicomplexa is a parasite living in the animal gut (43). Its presence on the skin may be due to fecal contamination since the pandas were housed in semi-closed enclosures.

## Conclusion

In this study, the skin mycobiota of healthy captive giant panda were profiled by 18S rRNA gene-based NGS sequencing. A total of 375 genera in 38 phyla were detected in samples from four seasons in a year, with Ascomycota, Basidiomycota, Streptophyta, and Chlorophyta as the dominant phyla taxa and *Trichosporon, Guehomyces, Davidiella, Chlorella, Asterotremella*, and *Klebsormidium* as the dominant genera. The results showed that skin mycobiota of the giant panda changed in different seasons, and that the diversity and abundance of the skin fungi were significantly higher in spring, summer, and autumn than in winter, which may have enhanced the opportunity to infect the host and contributed to the higher occurrence of dermatomycosis in fall and summer. Several known dermatomycosis-associated fungi were detected in the skin mycobiota of the healthy giant panda, indicating that dermatomycosis-associated fungi are the normal components of the skin mycobiota in the giant panda. The findings in this study uncovered the skin mycobiota of the captive giant panda and provided insights into the development of dermatophytosis.

## Data Availability Statement

The datasets presented in this study can be found in online repositories. The names of the repository/repositories and accession number(s) can be found below: NCBI SRA BioProject, Accession No: PRJNA610450.

## Ethics Statement

The animal study was reviewed and approved by Sichuan Agricultural University Animal Ethics Committee and China Conservation and Research Center for the Giant Panda Animal Ethics Committee.

## Author Contributions

XM, GL, YJ, MH, CW, YG, and SL carried out the sample collections, conceived the study, and drafted the manuscript. SC, YW, QZ, RW, and ZZu participated in the data analysis. ZZh and XM participated in the study design and coordination and helped draft the manuscript. All authors have read and approved the final manuscript.

## Funding

This study was supported by the Key Laboratory of National Forestry and Grassland Administration of Giant Panda National Park Open Fund (KLSFGAGP2020.023).

## Conflict of Interest

The authors declare that the research was conducted in the absence of any commercial or financial relationships that could be construed as a potential conflict of interest.

## Publisher's Note

All claims expressed in this article are solely those of the authors and do not necessarily represent those of their affiliated organizations, or those of the publisher, the editors and the reviewers. Any product that may be evaluated in this article, or claim that may be made by its manufacturer, is not guaranteed or endorsed by the publisher.

## Supplementary Material

Supplementary Figure 1 Rarefaction curves on the observed_otus indices to show that the sequencing depth is sufficient to represent the diversity in each sample.

Supplementary Table 1 The sample information (The same giant panda is marked in yellow).

Supplementary Table 2 The relative abundance of each OTU in each sample.
