# Supplementary figures and images for: Skin Mycobiota of the Captive Giant Panda (Ailuropoda melanoleuca) and the Distribution of Opportunistic Dermatomycosis-Associated Fungi in Different Seasons
Source: Front Vet Sci. 2021 Nov 4;8:708077. doi: 10.3389/fvets.2021.708077 (PMC8599956; doi:10.3389/fvets.2021.708077)

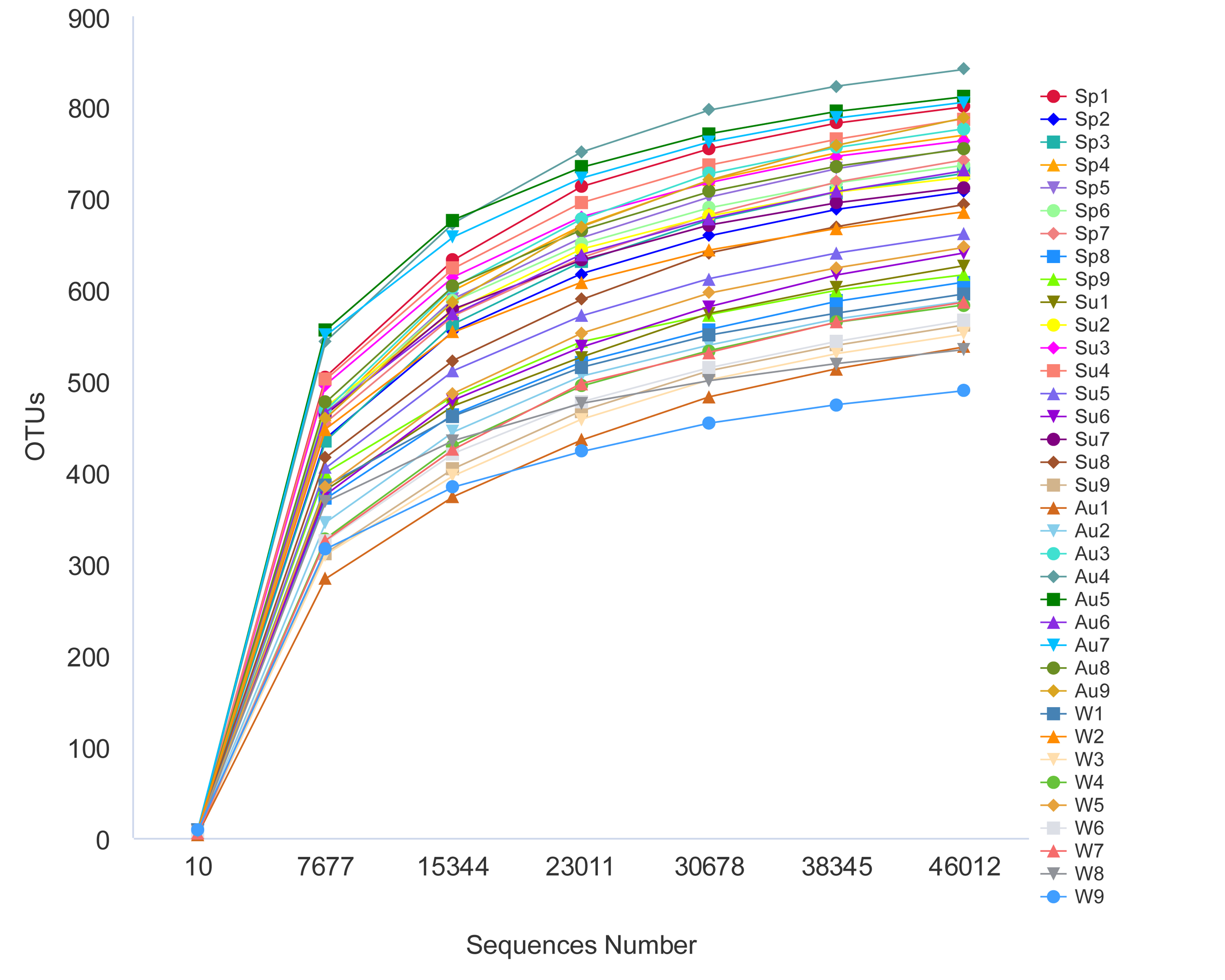

Supplement: Supplementary Figure 1 — Rarefaction curves on the observed_otus indices to show that the sequencing depth is sufficient to represent the diversity in each sample. [file Image_1.tif]
