# Supplementary material for: Skin Mycobiota of the Captive Giant Panda (Ailuropoda melanoleuca) and the Distribution of Opportunistic Dermatomycosis-Associated Fungi in Different Seasons
Source: Front Vet Sci. 2021 Nov 4;8:708077. doi: 10.3389/fvets.2021.708077 (PMC8599956; doi:10.3389/fvets.2021.708077)
Supplement: Supplementary Table 1 — The sample information (The same giant panda is marked in yellow). [file Table_1.DOCX]

**Table 1**

The sample information(The same giant panda is marked in yellow)

| Group | Sample number | sample name | Sampling time | Gender | date of birth |
| --- | --- | --- | --- | --- | --- |
| Winter | W1 | zhuang mei | 12.11.2014 | female | 07.26.2008 |
|  | W2 | feng yi | 12.11.2014 | male | 03.09.2015 |
|  | W3 | yuan yuan | 12.11.2014 | male | 08.23.1999 |
|  | W4 | wu jun | 12.11.2014 | male | 09.14.2007 |
|  | W5 | ge ge | 12.11.2014 | female | 09.06.2003 |
|  | W6 | bo yang | 12.11.2014 | male | 2004 |
|  | W7 | qian qian | 12.11.2014 | female | 08.13.1998 |
|  | W8 | shu qin | 12.11.2014 | female | 08.26.2009 |
|  | W9 | xi mei | 12.11.2014 | female | 08.08.2000 |
| Spring | Sp1 | zhuang mei | 03.11.2015 | female | 07.26.2008 |
|  | Sp2 | feng yi | 03.11.2015 | male | 03.09.2015 |
|  | Sp3 | yuan yuan | 03.11.2015 | male | 08.23.1999 |
|  | Sp4 | wu jun | 03.11.2015 | male | 09.14.2007 |
|  | Sp5 | ge ge | 03.11.2015 | female | 09.06.2003 |
|  | Sp6 | bo yang | 03.11.2015 | male | 2004 |
|  | Sp7 | qian qian | 03.11.2015 | female | 08.13.1998 |
|  | Sp8 | shu qin | 03.11.2015 | female | 08.26.2009 |
|  | Sp9 | xi mei | 03.11.2015 | female | 08.08.2000 |
| Summer | Su1 | zhuang mei | 06.11.2015 | female | 07.26.2008 |
|  | Su2 | feng yi | 06.11.2015 | male | 03.09.2015 |
|  | Su3 | yuan yuan | 06.11.2015 | male | 08.23.1999 |
|  | Su4 | wu jun | 06.11.2015 | male | 09.14.2007 |
|  | Su5 | ge ge | 06.11.2015 | female | 09.06.2003 |
|  | Su6 | bo yang | 06.11.2015 | male | 2004 |
|  | Su7 | qian qian | 06.11.2015 | female | 08.13.1998 |
|  | Su8 | shu qin | 06.11.2015 | female | 08.26.2009 |
|  | Su9 | xi mei | 06.11.2015 | female | 08.08.2000 |
| Autumn | Au1 | zhuang mei | 09.11.2015 | female | 07.26.2008 |
|  | Au2 | feng yi | 09.11.2015 | male | 03.09.2015 |
|  | Au3 | yuan yuan | 09.11.2015 | male | 08.23.1999 |
|  | Au4 | wu jun | 09.11.2015 | male | 09.14.2007 |
|  | Au5 | ge ge | 09.11.2015 | female | 09.06.2003 |
|  | Au6 | bo yang | 09.11.2015 | male | 2004 |
|  | Au7 | qian qian | 09.11.2015 | female | 08.13.1998 |
|  | Au8 | shu qin | 09.11.2015 | female | 08.26.2009 |
|  | Au9 | xi mei | 09.11.2015 | female | 08.08.2000 |
